# AT1R Regulates Macrophage Polarization Through YAP and Regulates Aortic Dissection Incidence

**DOI:** 10.3389/fphys.2021.644903

**Published:** 2021-07-09

**Authors:** Xinhao Wang, Hongpeng Zhang, Yangyang Ge, Long Cao, Yuan He, Guoyi Sun, Senhao Jia, Airong Ma, Jie Liu, Dan Rong, Wei Guo

**Affiliations:** ^1^Department of Vascular Surgery, First Medical Center of Chinese PLA General Hospital, Beijing, China; ^2^Research Platform for Minimally Invasive Cardiovascular Surgery, Beijing Key Laboratory, Beijing, China; ^3^Zibo Central Hospital, Zibo, China

**Keywords:** macrophage, YAP, inflammatory, polarization, adhesion

## Abstract

Aortic dissection (AD) is one of the most fatal cardiovascular emergency. At the anatomical level, AD occurs due to the formation of intimal tears. However, the molecular mechanism underlying this phenomenon remains unknown. Angiotensin II (Ang II) is a important effector in the development of cardiovascular disease that acts through binding to angiotensin type 1 receptor (AT1R). Yes-associated protein (YAP) was recently recognized as a key protein in macrophage activation. To determine whether AT1R and YAP are involved in macrophage-induced endothelial cell (EC) inflammation and AD incidence, we co-cultured THP-1 cells and HAECs in transwell chambers under different culture conditions and apply different conditions to the AD mice model. The results showed that Ang II promoted macrophage M1 polarization and adhesion, upregulated YAP phosphorylation, and induced EC injury that was related to increased levels of multiple pro-inflammatory chemokines. Blocking AT1R function pharmacologically or by transfection with AT1R siRNA can reduce the pro-inflammatory effect induced by Ang II. In addition, siRNA knock down of YAP expression further aggravated the pro-inflammatory effects of Ang II. Treatment with ARB effectively alleviated these pro-inflammatory effects. In the mice AD model, ARB effectively reduced the incidence of AD in mice, decreased M1 macrophages infiltration and AT1R content in the aortic wall and increased the tissue content of YAP. We found that AT1R induces YAP phosphorylation through binding to Ang II, and further promotes macrophage M1 polarization and adhesion to ECs. ARB reduces the incidence of AD in mice and affect macrophage polarization in mice aorta.

## Highlights

- Angiotensin II regulates YAP through angiotensin type I receptors in macrophages, and reduced aortic dissection incidence in mice.
- YAP affects macrophage polarization after being regulated by angiotensin type I receptor.

## Introduction

Aortic dissection (AD) is a lethal disease that causes a large number of aortic-related deaths (Hiratzka et al., 2010). Epidemiological surveys showed that the incidence of thoracic aortic dissection is 3–4 per 100,000 individuals per year (Olsson et al., 2006; Kochanek et al., 2011). AD is defined as blood flow that enters the aortic media through intimal tears, followed by formation of a true lumen (TL) and a false lumen (FL) with or without communication (Erbel et al., 2014). Clinically, AD can cause various serious complications, such as aortic rupture, visceral ischemia and etc. The majority of patients with non-operative ascending AD (Stanford type A) die within 2 weeks (Chen et al., 1997). Unfortunately, due to its unpredictable nature, little is known about pathological and molecular mechanisms that occur before or after onset of AD. Clinical treatment options for AD include optimal medical treatment (OMT), aortic replacement, and thoracic endovascular aorta repair (TEVAR) (Jia et al., 2013). However, the AD patients mortality rate after treatment remains high (Khayat et al., 2018). The surgical mortality for AD ranges from 10 to 35%, even at experienced medical centers (Nienaber and Clough, 2015). OMT is commonly used to treat AD in the clinic, mainly to protect the fragile aortic wall by reducing heart rate and blood pressure. However, there is no uniform standard for the selection of antihypertensive drugs, with most doctors relying on their own experience to select the most appropriate drugs. There have been no studies addressing which antihypertensive drugs are most effective in treating AD. Therefore, AD pathogenesis and the best treatment options are still poorly characterized.

Previous studies have shown that the occurrence of AD is associated with mutations in certain genes, such as *FBN1*, *COL3A1*, *TGBFR1*, and *ACTA2*. In addition to these genetic factors, there are other risk factors for AD such as hypertension, dyslipidemia, and smoking (Ohno-Urabe et al., 2018). Degeneration of the aortic media is now widely recognized as a histopathological risk factor for AD formation. This degeneration is caused by loss and disruption of elastic fibers and increased deposition of proteoglycans (Hiratzka et al., 2010; Kurihara et al., 2012). Degeneration only weakens the media layer, while most cases of AD begin with formation of an intimal tear (Erbel et al., 2014).

The occurrence of these non-genetic risk factors suggests that inflammation and intimal damage may contribute to the formation of intimal tears. Angiotensin II (Ang II) is the main effector peptide of the renin-angiotensin system, which can induce vasoconstriction, hypertrophy and extracellular remodeling through Ang II type 1 receptor (AT1R) (Schluter and Wenzel, 2008). Moreover, Ang II also regulates macrophage recruitment and polarization, which in turn triggers inflammation of local tissues (Yamamoto et al., 2011; Kaji, 2018). More importantly, studies have shown that Ang II contributes to the development of AD in humans and experimental animals (Daugherty et al., 2000). Angiotensin receptor blocker (ARB) is an antihypertensive drug that is commonly used to control blood pressure of AD patients through blocking AT1R. Therefore, we sought to determine whether ARB has other effects on AD in addition to lowering blood pressure and maintaining aortic wall stability.

Yes-associated protein (YAP) is a pluripotent intracellular connexin, which can act as a transcriptional co-activator and the main effector of the Hippo-YAP signaling pathway. Nuclear YAP binds to transcriptional enhancer associated domain transcription factors (TEADs) to affects cell proliferation and apoptosis through regulating transcriptional processes (Valis et al., 2011). Mammalian sterile 20-like kinase-1 and mammalian sterile 20-like kinase-2 (Mst1/2) act as the main mediators of the Hippo YAP pathway, and up-regulate YAP phosphorylation level. Since phosphorylated YAP (p-YAP) cannot enter the nucleus, it remains in the cytoplasm, so it cannot participate in regulating transcription (Ren et al., 2008). YAP plays a key role in mediating the proliferation, migration, apoptosis, and phenotypic transition of vascular smooth muscle cell (Feng et al., 2016). In addition, a recent study found that Hippo-YAP signaling in hepatocytes inhibits macrophage infiltration during formation of the protumoral microenvironment by inhibiting YAP, thereby maintaining normal liver growth (Kim et al., 2018). This prompted us to ask whether YAP plays a role in macrophage-induced endothelial cell (EC) inflammation and injury, and whether YAP is regulated by AT1R in macrophages. Therefore, the purpose of this study is to investigate the regulation of Ang II on AT1R-mediated YAP and the mechanism of macrophage-induced EC damage.

## Materials and Methods

### Animals

Forty-eight wild type mice (male, 22–24 g) were purchased from the experimental animal center of the Academy of Military Medical Sciences of PLA, Beijing, China. Twelve mice were randomly selected as the control group. The remaining 36 mice were given 1 g/kg β-aminopropionitrile (BAPN) (Xiya reagent, 151-18-8) water daily, and were randomly divided into three groups after 2 weeks of feeding. Group 1 continued BAPN feeding for 2 weeks; Group 2 and Group 3 continued BAPN feeding while telmisartan (T8949-10MG, Sigma-Aldrich, St. Louis, MO, United States) and nifedipine were fed separately for 2 weeks. The mice were fed with nifedipine (N7634-1G, Sigma-Aldrich, St. Louis, MO, United States) in order to set up an animal model with similar blood pressure levels to the telmisartan feeding group. The reason is that YAP is regulated by mechanical signals (Endlich et al., 2017), so we try to avoid the influence of different blood pressure level on YAP regulation by feeding with nifedipine. All mice were fed for 4 weeks, and each group of mice took Tail-cuff tail pressure method at five time points: 1 day before feeding, after feeding for 1, 2, 3, and 4 w to monitor changes in systolic blood pressure. Feeding mice with nifedipine and monitoring the molecular tail pressure is because that YAP is a pressure-sensitive protein. In order to eliminate the difference between the groups caused by the difference in aortic wall pressure, the blood pressure of the mice is controlled at the same level to observe the effect of ARB drugs on YAP.

### Cell Culture

Human monocytic THP-1cells (Shanghai Zhong Qiao Xin Zhou Biotechnology Co., Ltd., ZQ0086) were cultured in RPMI-1640 medium (Life Technologies, Carlsbad, CA, United States) supplemented with 10% FBS, 2 mM L-Glutamine, and 1% antibiotics (penicillin, streptomycin, and amphotericin-B) (Sigma-Aldrich, St. Louis, MO, United States). THP-1 were incubated with 100 mg/ml phorbol 12-myristate 13-acetate (PMA, Solarbio, P6741)for 48 h to induce differentiation into quiescent M0 macrophages. When the cells were adherent, they were transferred to PMA-free medium to obtain resting macrophages (M0). Then, M0 cells were seeded into the lower wells of transwell chamber with 0.4 μm pores (6 × 10^4^ cells/well) (Corning, Corning, NY, United States), and human aortic endothelial cells (HAECs) were seeded into the upper wells (5 × 10^5^ cells/well). Different treatment for M0 macrophages were placed in the lower wells after 24 h incubation as follows: Control group, THP-1 + HAECs only; group 1, THP-1 + HAECs with Ang II (1 μM) treatment; group 2, THP-1 + HAECs with Ang II (1 μM) and small interfering RNA (siRNA) negative control (NC) transfection; group 3, THP-1 + HAECs with Ang II (1 μM) and AT1R siRNA transfection; group 4, THP-1 + HAECs with Ang II (1 μM) and 1 μl DMSO; and group 5, THP-1 + HAECs with Ang II (1 μM) and the ARB 1 μl (20 mM) telmisartan treatment. After applying various intervention conditions, the cells were further cultured at 37°C and 5% CO_2_ for 72 h, and the total incubation time was 96 h.

### CCK-8 Assay and Flow Cytometric Analysis

THP-1 and HAECs were inoculated into multi-well plates (5 × 10^3^ cells/well). Cells were inoculated with three replicate sets per experimental group. We followed the CCK-8 method in our previous study (Wang et al., 2020).

### ELISA for ET-1, IL-6 and MMP-9 Levels in the Supernatant

Endothelin (ET)-1 levels in supernatant from each group at the end of the 96 h treatment and in the mice serum from different group after 4 week feed were tested through competitive inhibition ELISA with ET-1 Antibody ELISA kit (USCN Life Sciences, Inc.), and interleukin (IL)-6 and matrix metallopeptidase (MMP)-9 in the cell solutions and mice serum were detected by double antibody ELISA with IL-6 ELISA Kit (USCN Life Sciences, Inc.) and MMP9 ELISA Kit (USCN Life Sciences, Inc.), respectively. We follow our previous research for ELISA (Wang et al., 2020).

### Flow Cytometric Analysis of Macrophage Polarization

The macrophages were collected from the lower chamber for each group, counted, resuspended, and the cell number was adjusted to approximately 1 × 10^5^ per sample. Fluorescent antibodies were diluted with FACS buffer at the following ratios: anti-CD86-PE (1:200, BioLegend, Inc., 305406), anti-CD68-FITC (1:100, BioLegend, Inc., 305206), and anti-CD206-APC (1:200, BioLegend, Inc., 321110). Fifty microliters of each diluted antibody working solution was added to each cell sample, after which the cells were incubated at 4°C for 30 min in the dark. After incubation, 200 μl of FACS buffer was added to each sample. The mixture was centrifuged at 1,000 rpm for 10 min at 10°C, and the supernatant was discarded. Cell samples were analyzed using a FACS Calibur flow cytometer and Cell Quest software (Becton Dickinson, Franklin Lakes, NJ, United States).

### Western Blotting forAT1R and YAP

Each group of THP-1 after 96 h co-culture and AD mice aorta tissue was collected for Western blotting test. Membranes were subsequently incubated overnight at 4°C with the following primary antibodies: Anti-YAP (cat. no. AF6328; polyclonal, rabbit anti-human and mouse; 1:1,000; Affinity Biosciences), anti-p-YAP (Ser127) (cat. no. AF3328; polyclonal, rabbit anti-human and mouse; 1:1,000; Affinity Biosciences), anti-AT1R (cat. no. DF4910; polyclonal, rabbit anti-human and mouse; 1:1,000; Affinity Biosciences), anti-GAPDH (cat. no. ab9485; polyclonal, rabbit anti-human and mouse; 1:2,500; Abcam). The specific operations and reagents are the same as our previous research (Wang et al., 2020). ImageJ (V1.8.0, National Institutes of Health) was used to evaluate the density of bands by quantitative densitometry.

### AT1R siRNA and YAP siRNA Transfection

Shanghai GenePharma Co., Ltd., designed and synthesized AT1R siRNA as previous study (Wang et al., 2020). YAP siRNA was also designed and synthesized be the same company as follows: YAP – 860, GCAUCUUCGACAGUCUUCUTT; YAP – 941, GGUCAGAGAUACUUCUUAATT; YAP – 1662, GGUGAUACUAUCAACCAAATT; NC, UUCUCCGAACGUG UCACGUTT. AT1R siRNAs and YAP siRNA were transfected into cells with Lipofectamine^®^ 2000 (Thermo Fisher Scientific, Waltham, MA, United States) following the manufacturer’s protocol. M0 THP-1cells were inoculated into a 6-well plate at 5 × 10^5^ cells/well, and cultured overnight at 37°C in 5% CO_2_ incubator. Two hours before transfection, they were mixed with serum-free MEMα medium (Gibco; Thermo Fisher Scientific).

### Reverse Transcription-Quantitative PCR (RT-qPCR)

RT-qPCR for siRNA selection: At 48 h after siRNA transfection, as described in the previous section, AT1R relative and YAP relative mRNA were collected from the transfected THP-1 cells. The RT-qPCR was performed as the same way as our previous study (Wang et al., 2020). Based on the effectiveness of AT1R siRNA shown in Figure 4A, SiRNA-165 was used in subsequent experiments. And, siRNA-1662 was chosen to knockdown YAP which is exhibit in Figure 6A. RT-qPCR for CTGF and CYR61: After 96 h co-culture, CTGF and CYR61 were collected from different group of THP-1. RevertAid First Strand cDNA Synthesis kit (Lot:U9126, TIANGEN, Inc.) were used according to the manufacturer’s protocol in order to reverse transcribe 1 μg RNA into first-strand cDNA. FastStart Universal SYBR Green Master (Rox; 04913914001, Hoffmann-La Roche, Ltd.) was then used to perform quantitative PCR as follows: 2 min at 50°C, 10 min at 95°C, and then 45 cycles of 30 s at 95°C and 30 s at 60°C. Amplification was performed on a PCR System 7300 (Applied Biosystems; Thermo Fisher Scientific, Inc.). The 2−^ΔΔ^ Cq method was used to analyze the results.

### Macrophage Adhesion Assay

M0 macrophages were labeled with calcein acetoxymethyl ester (MedChemExpress, HY-D0041) and incubated with HAECs at a density of 2 × 10^5^ for 2 h. Depending on the experimental group, the cells were then transfected with the AT1R siRNA for 48 h or treated with Ang II and telmisartan for 24 h. Then, the cells were lightly rinsed with 1% BSA in PBS to remove unadhered cells. All cells were labeled with DAPI, and the proportion of macrophages adhered to each group was analyzed by fluorescence microscopy. The proportion of adherent cells (%) = the number of macrophages (green fluorescence)/the total number of cells (blue fluorescence) × 100.

### Histological and Immunocytochemical Characterization of AD Tissue

The mice aortas were separated and fixed in the 4% paraformaldehyde for 48 h after 4 w of treatment with different drugs. Serial paraffin cross section (5 μm) of mice aorta were prepared for histological analysis. Aorta sections were stained with eosin and hematoxylin for morphological assessment. Immunostaining of CD68 and polarization markers (CD86, CD86, 1:1,000, Santa Cruz, CA, United States) was performed to detect macrophages infiltration in mice aorta. The images of immunofluorescence were digitally captured on a Carl Zeiss LSM880 laser scanning confocal microscope (Carl zeiss, Jena, Germany). CD68 + cell number or average integral optical density of CD 86 and CD 206 in the mice aortic wall were quantitative analyzed from 10 samples per group using Image Pro Plus 6.0 software (Media Cybernetics, Silver Spring, MD, United States).

### Statistical Analysis

Statistical analysis was performed using SPSS software (v19.0; IBM Corp.) to evaluate differences among groups. Comparisons among >2 groups were performed using one-way ANOVA followed by Tukey’s *post hoc* test. Data are presented as mean ± standard deviation, and *P* < 0.05 was considered to indicate a statistically significant difference. All experiments were repeated in triplicates (Wang et al., 2020).

## Results

### Ang II Reduces Macrophage and EC Proliferation and Triggers an Inflammatory Response, and Treatment With ARB Suppresses These Effects

**FIGURE 1:**
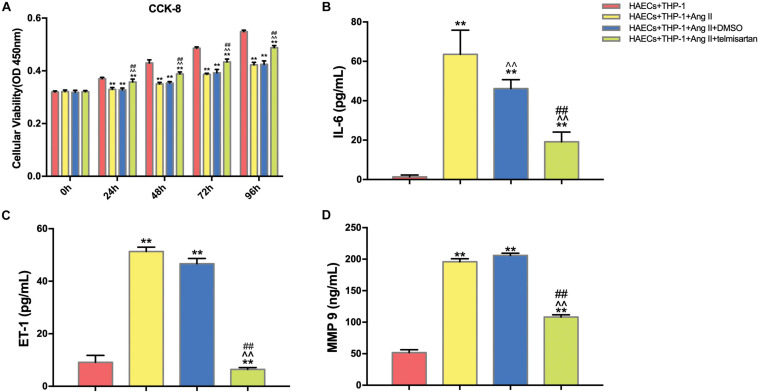
Ang II inhibits cell proliferation and promotes ECs inflammation in co-cultured HAECs and THP-1 cells, and telmisartan counteracts this effect. **(A)** M0 macrophages were first co-cultured with HAECs for 24 h, and then the intervention conditions of each group were added to the lower chamber and continued to co-culture for 72 h. CCK-8 detection of cell proliferation in THP-1 cell and HAEC co-cultures at 0, 24, 48, 72, and 96 h. After 96 h of co-culture, Ang II (1 μM, 24 h) significantly decreased the proliferation of HAECs and THP-1 cells (*P* < 0.01), and this phenomenon was significantly reduced by telmisartan treatment (20 μM, 24 h) (*P* < 0.01); **(B)** ELISA results of IL-6 in supernatants from different samples after 96 h co-culture; **(C)** ELISA results of ET-1 in supernatants from different samples after 96 h co-culture; **(D)** ELISA results of MMP-9 in supernatants from different samples after 96 h co-culture. Ang II (1 μM, 24 h) significantly increased the expression of IL-6, ET-1, and MMP 9 in the supernatants compared with the control group (which was not treated with Ang II). IL-6, ET-1, and MMP-9 levels were significantly lower in the telmisartan (20 μM, 24 h) group compared with the DMSO group; (*n* = 3, mean and S.D., *t*-test) **P < 0.05 compared with the HAECs + THP-1 group; ^∧∧^*P* < 0.05 compared with the HAECs + THP-1 + Ang II group; ^##^*P* < 0.05 compared with the HAECs + THP-1 + DMSO group.

We first divided the co-cultured cells (THP-1 cells and HAECs) into four groups: control group, co-cultured cells only; group 1, co-cultured cells with Ang II; group 2, co-cultured cells with Ang II and DMSO; group 3, co-cultured cells with Ang II and telmisartan (an ARB); group 4, co-cultured cells with Ang II and nifedipine (a calcium channel blocker). Then, we tested the proliferation in each group using a CCK-8 assay (Figure 1A). After Ang II treatment, the cell proliferation activity was significantly lower than the control group (*P* < 0.01). Compared with the control group, cell proliferation was significantly increased after treatment with telmisartan (group 3) (*P* < 0.01). Based on these results, we speculated that Ang II damages HAECs, and that this damage is likely due to Ang II–mediated promotion of macrophage infiltration and adhesion, which cause an inflammatory reaction. Ang II is known to upregulate ET-1, IL-6 and MMP-9 to promote endothelial inflammation (Nurden, 2011; Pacurari et al., 2014). Therefore, we measured ET-1, IL-6, and MMP-9 levels in supernatants from each group (Figures 1B–D), and found that the circulating levels of these proteins were all significantly higher in group 1 and group 2 than in the control group and group 3 (*P* < 0.05). These findings indicate that Ang II promotes ECs inflammation and that ARB can alleviate these pro-inflammatory effects.

### Ang II Promotes Macrophage M1 Polarization and Adhesion, and Treatment With ARB Suppresses These Effects

**FIGURE 2:**
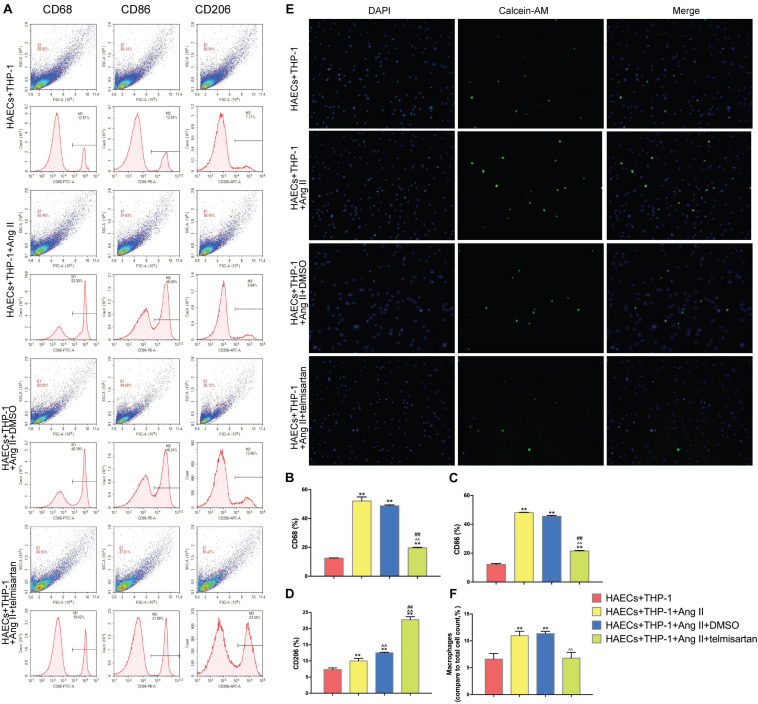
Ang II promotes THP-1 polarization to a CD68 + CD86 + phenotype and increases THP-1 adhesion. Telmisartan attenuates these effects and promotes THP-1 polarization to a CD206 + phenotype. **(A)** Flow cytometry analysis of CD68, CD86, and CD206 expression in the different groups after 96 h co-culture. The data shown in the one-factor histogram for each group represent the cells in the red circle in the upper scatter plot. HAECs + THP-1 group: CD68, 12.51%; CD86, 12.54%; CD206, 7.11%. HAECs + THP-1 + Ang II (1 μM, 24 h) group: CD68, 53.30%; CD86, 48.09%; CD206, 9.64%. HAECs + THP-1 + Ang II (1 μM, 24 h) + DMSO (20 μM, 24 h) group: CD68, 48.19%; CD86, 46.24%; CD206, 12.66%. HAECs + THP-1 + Ang II + telmisartan (20 μM, 24 h) group: CD68, 19.42%; CD86, 21.89%; CD206, 23.34%; **(B,C)** The proportion of CD68 + and CD86 + cells, respectively [*n* = 3, mean and Standard Deviation (S.D.), *t*-test, **P < 0.05 compared with the HAECs + THP-1 group; ^∧∧^*P* < 0.05 compared with the HAECs + THP-1 + Ang II group; ^##^*P* < 0.05 compared with the HAECs + THP-1 + DMSO group]. The proportion of CD68 + and CD86 + cells was significantly increased by treatment with Ang II (*P* < 0.01), and telmisartan reversed this increase. **(D)** The proportion of CD206 + cells (*n* = 3, mean and S.D., *t*-test, symbols are the same as in **B,C**). The proportion of CD206 + cells increased in response to treatment with Ang II (*P* < 0.05), and telmisartan strongly promoted THP-1 polarization to a CD206 + phenotype (*P* < 0.01); **(E)** Fluorescence staining of adherent macrophages in the different groups. All cells were labeled with DAPI (blue), and the number of adherent macrophages increased significantly in response to treatment with Ang II. This effect was not seen in cells treated with telmisartan; **(F)** The ratio between the number of macrophages and the total number of cells (*n* = 3, mean and S.D., *t*-test, symbols are the same as in **B,C**).

Pro-inflammatory M1 (CD68 +, CD86 +) macrophage polarization can cause acute inflammation, while M2 (CD206 +) polarization is associated with cell proliferation and fibrosis (Guo et al., 2017). Therefore, the proportion of M1 cells was assayed to determine the severity of inflammation in each group. To do this, we analyzed the proportion of CD68 +, CD86 +, and CD206 + cells in the lower chamber of each sample by flow cytometry (Figure 2A). Compared with the control group, Ang II had a positive effect on M1 polarization in group 1 and group 2 (*P* < 0.05). However, the proportion of M1 cells was significantly decreased (*P* < 0.01), while the proportion of CD206 + cells was significantly increased (*P* < 0.01) after treatment with telmisartan in group 3 (Figures 2B–D). This led us to hypothesize that the higher number of pro-inflammatory macrophages present causes more macrophages to adhere to endothelial cells, producing inflammation and intimal damage (Ding et al., 2016). To verify this hypothesis, we examined the adhesion rate of THP-1 cells to HAECs by immunocytochemistry (Figure 2E). As expected, the results showed that the adhesion rate of THP-1 cells in group 1 and group 2, which contained Ang II, was significantly higher than that of the control group (Figure 2F). This increase in adhesion rate due to Ang II was reduced in group 3 due to treatment with ARB. This suggests that Ang II promotes the development of an inflammatory response and intimal injury, while treatment with ARB can alleviate these effects and may promote reconstruction of the disrupted tissue by increasing the proportion of M2 cells (Ivanova et al., 2019).

### Ang II Reduces the YAP/p-YAP Ratio of Macrophages, and ARB Treatment Can Reverse These Effects

**FIGURE 3:**
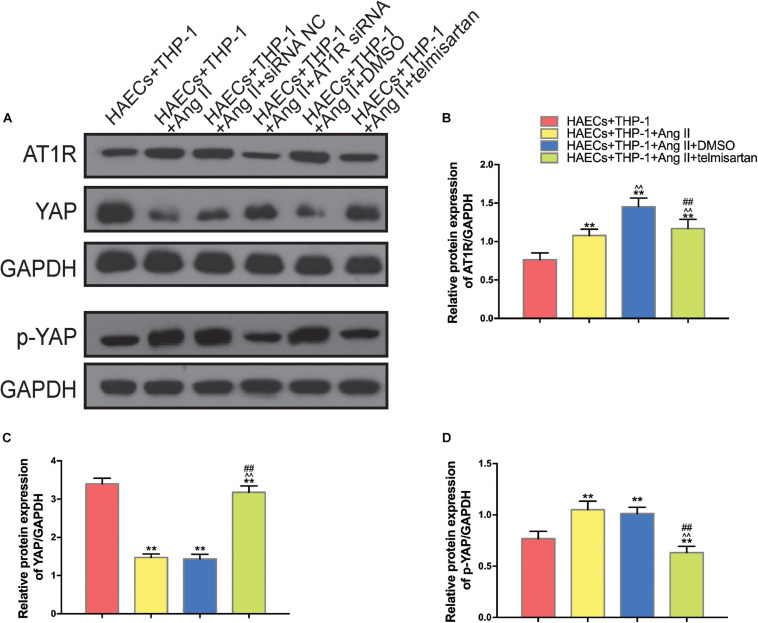
Ang II promotes AT1R expression and YAP phosphorylation, and treatment with telmisartan or transfection with AT1R siRNA counteract this effect. **(A)** Western bolt analysis of six independent lysates from 96 h co-cultured HAECS + THP-1 cells that were untreated, treated with Ang II, treated with Ang II and siRNA NC, treated with Ang II and AT1R siRNA, treated with Ang II and DMSO, or treated with Ang II and telmisartan. Ang II treatment up-regulated the content of AT1R and p-YAP, and this effect was alleviated by AT1R siRNA transfection or telmisartan treatment. **(B–D)** The ratio between AT1R and GAPDH, YAP and GAPDH, p-YAP and GAPDH or p-YAP and YAP expression (*n* = 3, mean and S.D., *t*-test, **P < 0.05 compared with the HAECs + THP-1 group; ^∧∧^*P* < 0.05 compared with the HAECs + THP-1 + Ang II group; ^##^*P* < 0.05 compared with the HAECs + THP-1 + DMSO group).

In order to identify the changes in the expression levels of YAP and AT1R in different groups, we used western blotting to detect the expression of AT1R, YAP, and p-YAP in each group of THP-1 cells (Figure 3A). Compared with the control group, YAP expression was decreased in THP-1 cells from group 1 and group 2, which were treated with Ang II (Figure 3C), while AT1R (Figure 3B), and p-YAP (Figure 3D) expression were increased in these groups (*P* < 0.05). In group 3, treatment with telmisartan alleviated these changes in expression (Figures 3B–D). These data indicate that Ang II promotes YAP phosphorylation, preventing it from entering the nucleus. Next, we used siRNA transfection to provide further evidence for the role of AT1R in this process.

### AT1R Knockdown and Treatment With ARB Drugs Have the Same Effect on Ang II- Mediated Regulation of Macrophages

**FIGURE 4:**
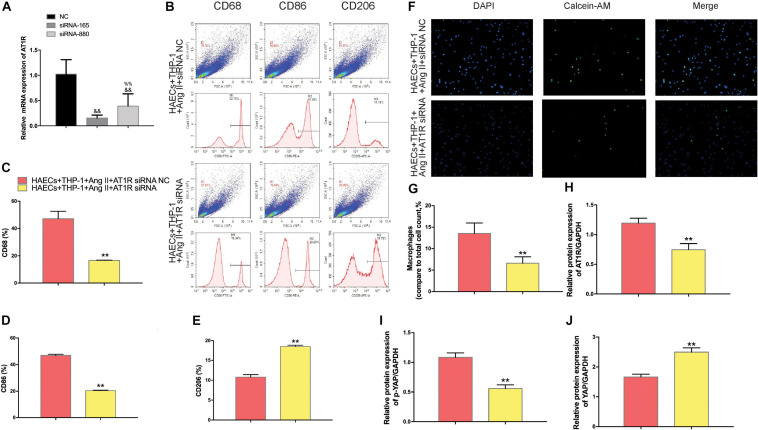
The effect of AT1R siRNA transfection on macrophages was consistent with the effects of telmisartan treatment, further demonstrating that Ang II regulates macrophage polarization and adhesion activity by binding to AT1R. **(A)** AT1R siRNA knockdown efficiencies (*n* = 3, mean and S.D., *t*-test, ^&&^*P* < 0.05 compared with siRNA NC;^%%^*P* < 0.05 compared with siRNA-165); **(B)** Flow cytometry analysis of CD68, CD86, and CD206 expression after 96 h co-culture in both groups. The data shown in the one-factor histogram for each group represents the cells in the red circle in the upper scatter plot. HAECs + THP-1 + Ang II (1 μM, 24 h) + siRNA NC group: CD68, 52.79%; CD86, 47.84%; CD206, 11.15%. HAECs + THP-1 + Ang II + AT1R siRNA group: CD68, 16.34%; CD86, 20.69%; CD206, 18.79%; **(C–E)** The proportion of CD68 +, CD86 +, and CD206 + cells, respectively (*n* = 3, mean and S.D., *t*-test, **P < 0.05 compared with the HAECs + THP-1 + siRNA NC group); **(F)** Fluorescence staining of adherent macrophages after AT1R siRNA transfection. All cells were labeled with DAPI (blue), and the number of adherent macrophages decreased significantly with AT1R siRNA transfection; **(G)** The ratio between the number of macrophages and the total number of cells (*n* = 3, mean and S.D., *t*-test, symbols are the same as in **C,D,E**); **(H–J)**, The ratio between AT1R and GAPDH, p-YAP and YAP, or YAP and GAPDH expression was calculated based on the western blot results shown in Figure 3A (*n* = 3, mean and S.D., *t*-test, symbols are the same as in **C–E**).

**FIGURE 5:**
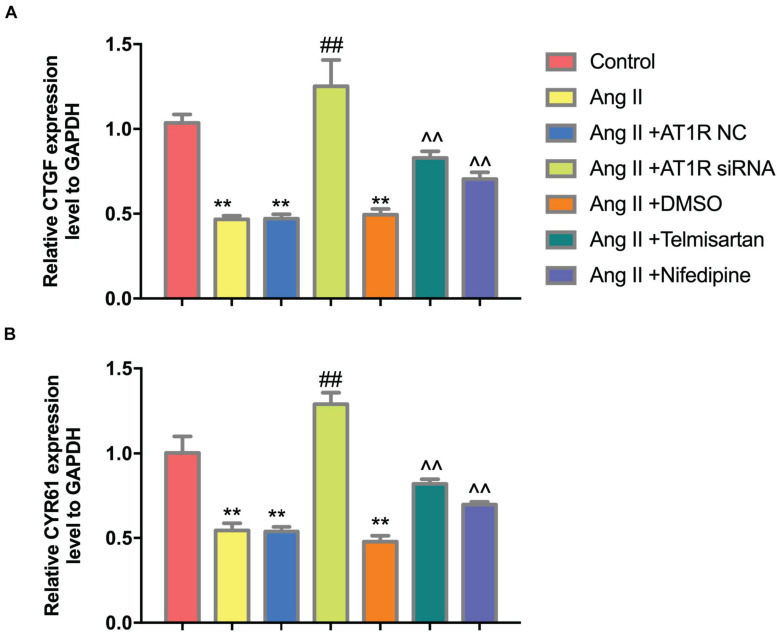
Ang II can reduce the expression of CTGF and CYR61, while AT1R siRNA transfection and Telmisartan treatment can alleviate this effect. **(A)** The qRT-PCR results of CTGF in THP-1 from different group after 96 h co-culture; **(B)** The qRT-PCR results of CYR61 in THP-1 from different group after 96 h co-culture. (*n* = 3, mean and S.D., *t*-test, **P < 0.05 compared with the control group; ^##^*P* < 0.05 compared with the HAECs + THP-1 + Ang II group; ^∧^^∧^*P* < 0.05 compared with the HAECs + THP-1 + Ang II + DMSO group).

To further confirm that Ang II regulation of macrophages is mediated by AT1R, we used an siRNA to knock down AT1R expression. RT-PCR analysis was performed to select the optimal AT1R siRNA, AT1R siRNA-165 (Figure 4A). The cells were divided into two groups: THP-1 cells + HAECs + Ang II + control siRNA and THP-1 cells + HAECs + Ang II + AT1R siRNA. Then, we tested cell proliferation, macrophage polarization, macrophage adhesion, and YAP phosphorylation as described above. THP-1 M1 polarization and adhesion were inhibited by AT1R knockdown, and M2 polarization was upregulated (Figures 4B–G). Transfection with the AT1R siRNA had almost the same effect as treatment with ARB on the consequences of treatment with Ang II, which provides more evidence that the pro-inflammatory effects of Ang II are mediated by AT1R (Figures 4H–J). Next, we asked whether there is a link between the regulated macrophage polarization and changes in YAP phosphorylation triggered by Ang II; or in other words, whether Ang II regulates macrophage polarization through phosphorylation of YAP. To test the effect of AT1R on YAP activity, we tested the expression of YAP-related genes CTGF and CYR61. As shown in Figure 5, Ang II can cause the down-regulation of CTGF and CYR61, while AT1R siRNA and Telmisartan treatment can alleviate this phenomenon.

### YAP Knockdown Impairs Ang II Regulation of Macrophage Polarization and Adhesion

**FIGURE 6:**
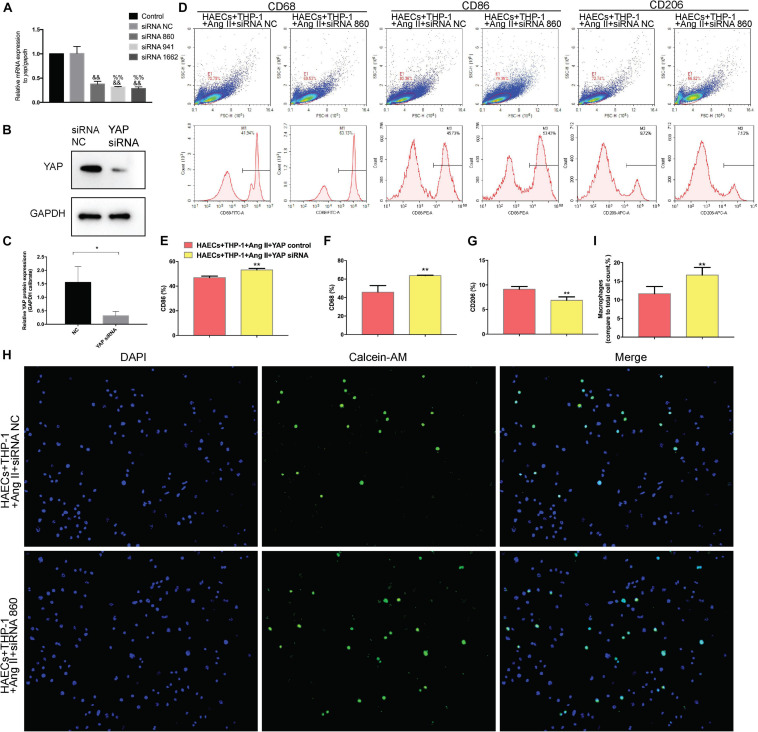
Transfecting macrophages with a YAP siRNA further enhanced Ang II–induced macrophage M1 polarization and adhesion. **(A)** YAP siRNA efficiencies (*n* = 3, mean and S.D., *t*-test, ^&&^*P* < 0.05 compared with siRNA NC;^%%^*P* < 0.05 compared with siRNA 860); **(B,C)** Western Blot of YAP after siRNA transfection *P < 0.05 compared with siRNA NC; **(D)** Flow cytometry analysis of CD68, CD86, and CD206 expression after 96 h co-culture in both groups. The data in the one-factor histogram represents the cells in the red circle in the upper scatter plot. HAECs + THP-1 + Ang II (1 μM, 24 h) + siRNA NC group: CD68, 41.94%; CD86, 45.73%; CD206, 9.72%. HAECs + THP-1 + Ang II + YAP siRNA group: CD68, 63.13%; CD86, 53.42%; CD206, 7.13%; **(E–G)** The proportion of CD68 +, CD86 +, and CD206 + cells, respectively (*n* = 3, mean and S.D., *t*-test, **P < 0.05 compared with the HAECs + THP-1 + siRNA NC group); **(H)** Fluorescence staining of adherent macrophages after YAP siRNA transfection. All cells were labeled with DAPI (blue), and the number of adherent macrophages was significantly decreased after transfection with the AT1R siRNA; **(I)** The ratio between the number of macrophages and the total number of cells (*n* = 3, mean and S.D., *t*-test, symbols are the same as in **C,D,E**).

To determine whether YAP is a key mediator of Ang II regulation of macrophage polarization and adhesion, we knocked down YAP using an siRNA. To select the optimal siRNA for knocking down YAP expression, we tested the effect of different YAP siRNAs by RT-PCR. The RT-PCR results showed that the effect of YAP siRNA860 was the most stable (Figure 6A). Next, we established two groups: THP-1 cells + HAECs + Ang II + control siRNA and THP-1 cells + HAECs + Ang II + YAP siRNA. The cells were cultured as previously, after which THP-1 polarization and adhesion in each group were detected by flow cytometry (Figure 6D) and immunocytochemistry (Figure 6H), respectively. The results showed that siRNA knockdown of YAP increased THP-1 M1 polarization (Figure 6B–E) and increased the Ang II-induced macrophages adhesion rate (Figures 6F,G). Also, the ratio between the number of macrophages and total number of cells was higher after YAP knockdown (Figure 6I). Taken together, these findings indicate that Ang II regulates pro-inflammatory polarization and adhesion in macrophages by altering YAP expression.

### ARB Can Effectively Reduce the Incidence of Dissection, and Significantly Reduce Macrophage Infiltration and Polarize It to M2

The mice in each group received the aortic anatomy surgery after 48 days of feeding. Blood pressure levels of mice in groups 2 and 3 remained similar throughout the entire breeding process (relevant data is in the supplement). The incidence of AD in each group was 0/12 (group control), 10/12 (group 1), 5/12 (group 2), and 8/12 (group 3), respectively. Two mice in group 1, one in group 2 and group 3 each died during feeding time, and these mice were presented AD after aortic anatomy surgery. The incidence of AD in group 2 was the lowest, and that in group 3 was also lower than that in group 1. The aorta specimens of mice in each group are shown in (Figures 7-A, A), A HE staining was performed on the paraffin-embedded sections of the aortic specimens of each group (Figures 7-A,B). The black arrow was the ECs and the red arrow was the macrophages. From the staining results, it can be seen that compared with the control group, the morphology of aortic intima ECs in AD model mice was destroyed, macrophages infiltration occurred, aortic adventitia teared formed a false lumen, and a large amount of inflammation occurred around the false lumen. Compared with AD mice, after treatment with telmisartan, the morphology of mouse aortic intima ECs recovered, inflammatory cell infiltration decreased. And, some of the macrophages still infiltrated in the aorta of AD mice treated with nifedipine, the outer membrane structure scattered. Figures 7-A,C,D shows the difference in macrophage differentiation by aortic immunofluorescence in each group of mice. The double markers CD68 and CD86 represent M1 macrophages, and the double markers CD68 and CD206 represent M2 macrophages. Compared with the control group, the number of aortic macrophages in the AD model group increased, the macrophage was mainly M1 type, and the M2 type macrophages were less observed. Compared with the AD model group, after treatment with telmisartan, the proportion of M1 type macrophages in the infiltrated area decreased, and the proportion of M2 type macrophage increased. Nifedipine has the same trend of effect, but the effect is lighter than telmisartan (Figures 7-B, A,B).

**FIGURE 7A:**
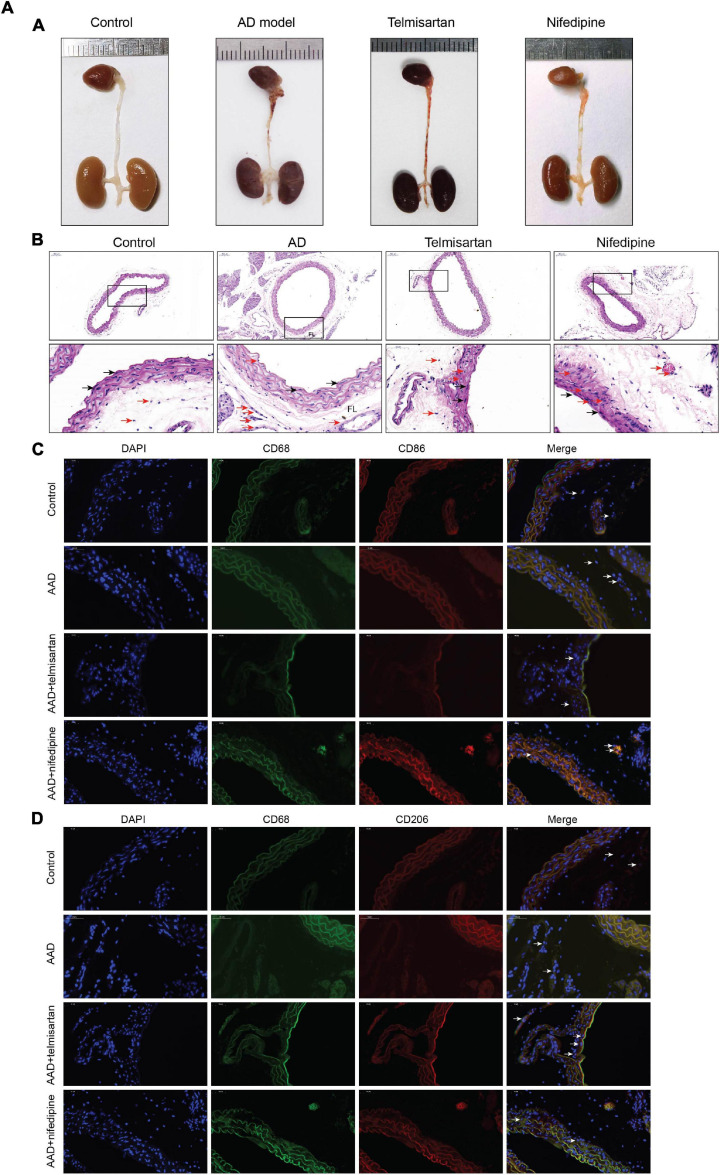
M1 macrophages infiltrate in the torn aortic wall in AD mice, and telmisartan treatment can increase M2 infiltration in the aortic wall. **(A)** Different groups of mouse aortic samples were presented. **(B)** HE staining of aortic tissue sections in each group. The first row is a magnified image of 100 times, and the second row is a magnified image of 400 times. The black arrow indicates the endothelial cells, and the red arrow indicates the macrophages. **(C)** 400-fold magnification of M1 macrophages (CD68 + /CD86 +) tissue fluorescent staining images. White arrow indicates M1 macrophages. **(D)** 400-fold magnification of M2 macrophages (CD68 + /CD206 +) tissue fluorescent staining images. White arrow indicates M2 macrophages.

**FIGURE 7B:**
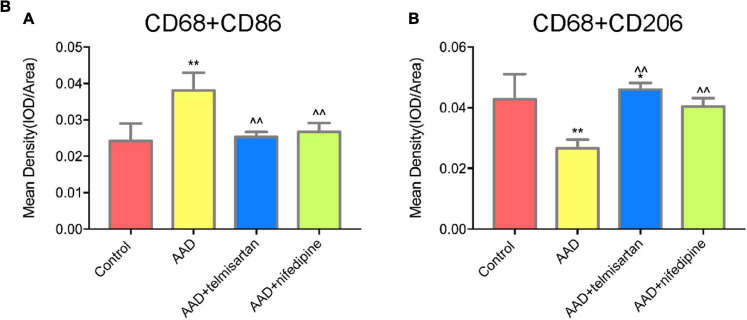
Quantification of tissue fluorescent staining images. **(A)** Quantification of M1 macrophages, ^∗∗^, ∧∧” specified in Figure 7-B. (CD68 + /CD86 +) tissue fluorescent staining images. **(B)** Quantification of M2 macrophages (CD68 + /CD206 +) tissue fluorescent staining images. *P < 005 compared with control group; **P < 0.01 compared with control group; ^^P < 0.05 compared with AAD group.

### The Serum Levels of Inflammatory Factors in AD Mice Are Increased, and Telmisartan Treatment Can Alleviated It

**FIGURE 8:**
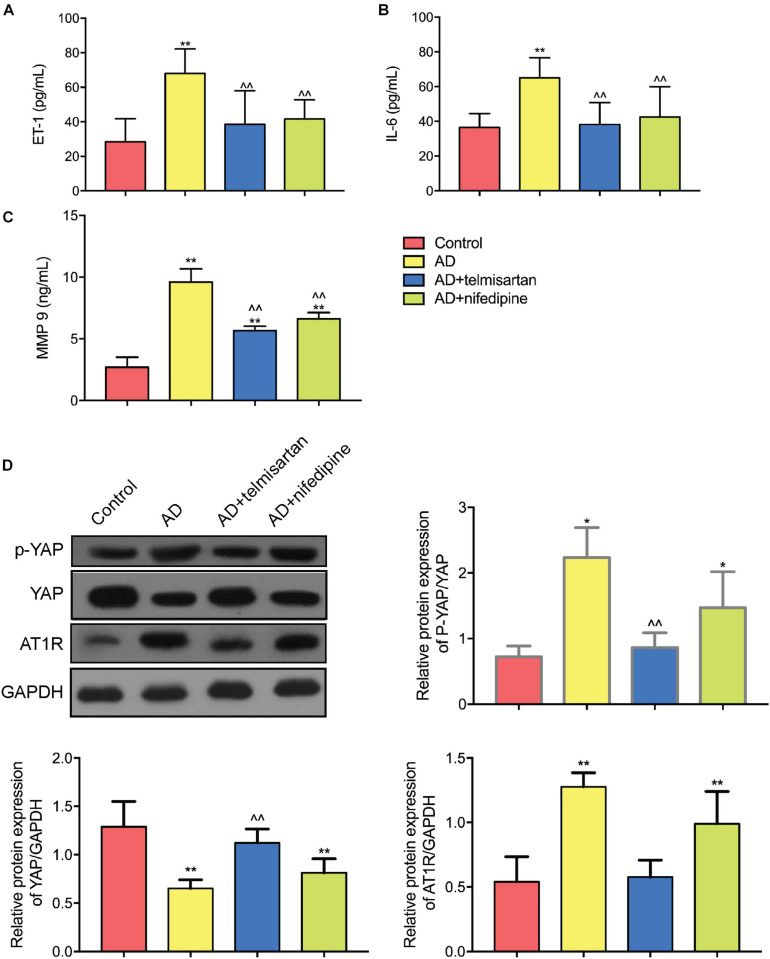
ELISA and Western Blot results of aortic tissue in each group of mice. **(A)** ET-1 ELISA results were shown. Both telmisartan and nifedipine treatments can effectively reduce ET-1 serum levels in the AD mice model. Telmisartan is slightly better than nifedipine. **(B)** IL-6 ELISA results were shown. Both telmisartan and nifedipine treatments can effectively reduce IL-6 serum levels in the AD mice model. Telmisartan is slightly better than nifedipine. **(C)** MMP 9 ELISA results were shown. Both telmisartan and nifedipine treatments can effectively reduce MMP 9 serum levels in the AD mice model. Telmisartan is slightly better than nifedipine. **(D)** Western Blot results of YAP and AT1R content in aortic tissue of each group were demonstrated. The contents of AT1R and p-YAP in the aortic tissue of AD group were significantly increased, and the total content of YAP was decreased. Telmisartan treatment can effectively alleviate this phenomenon, and nifedipine cannot affect the expression of AT1R and YAP. (*n* = 3, mean and S.D., *t*-test, *P < 0.01 compared with control group; **P < 0.05 compared with the control group; ^∧∧^*P* < 0.05 compared with the AD group; ^##^*P* < 0.05 compared with the AD + telmisartan group).

Figures 8A–C shows the expression levels of serum ET-1, IL-6, and MMP 9 in each group of mice. The expression levels of ET-1, IL-6, and MMP9 in AD model mice were increased compared with control group. Compared with AAD model mice, the expression levels of serum ET-1, IL-6, and MMP 9 were decreased in mice treated with telmisartan, and also the expression levels of serum ET-1, IL-6, and MMP9 in mice after treatment with nifedipine were decreased. The telmisartan treatment group had lower levels of ET-1, IL-6, and MMP9 in the serum compared with the nifedipine group. The results of the experiment showed that telmisartan and nifedipine treatment can reduce the expression of serum ET-1, IL-6, and MMP 9, and the anti-inflammatory effect of telmisartan is better than that of nifedipine.

### YAP/p-YAP Ratio Was Decreased in AD Mice, and Treatment With ARB Reverses These Effects While Treatment With Nifedipine Didn’t

Figure 8D shows the Western Blot test results for each group of aortic tissues. The results showed that the YAP/p-YAP ratio of AD mice was decreased and the expression of AT1R protein was increased compared with the control mice. Compared with the AD model group mice, the YAP/p-YAP ratio and the AT1R protein expression were decreased in the mice treated with telmisartan. Compared with AAD model mice, treatment of AD mice with nifedipine had no significant effect on the expression of YAP, p-YAP, and AT1R proteins. From this result and the results of previous cell experiments, we can speculate that the ARB drug telmisartan can inhibit the expression of AT1R in the aortic tissue of the AAD model and reduce the phosphorylation level of YAP protein.

## Discussion

In this study, we showed that Ang II binding to AT1R activates YAP phosphorylation, upregulates M1 polarization, and promotes macrophage adhesion to ECs. Based on assessing biomarkers for inflammation, we found that Ang II can trigger endothelial cell inflammation by upregulating YAP phosphorylation. In addition, we showed that blocking AT1R can inhibit macrophage M1 polarization, reduce macrophage adhesion, and downregulate YAP phosphorylation. YAP knock down inhibited Ang II regulation of macrophages. Therefore, our findings confirm that Ang II binding to AT1R upregulates YAP phosphorylation, which promotes macrophage M1 polarization and adhesion, thus triggering the endothelial cell inflammatory response. At the same time, we found in animal experiments that serum inflammatory factors increased in AD mice, M1 macrophage infiltration in the aortic wall of the dissection, and the ratio of YAP/p-YAP in the tissue decreased. Telmisartan treatment can effectively alleviate the above phenomenon and effectively reduce the incidence of AD. This suggests that the AT1R-YAP pathway may play an important role in macrophage-guided intimal inflammation and contribute to AD formation. However, the specific signaling mechanism remains to be determined. Future work will explore whether the classic Hippo pathway is involved.

This study shows that Ang II can activate YAP phosphorylation by binding to AT1R, and that transfection with AT1R siRNA or treatment with the telmisartan can inhibit Ang II-induced YAP phosphorylation, which may trigger the intracellular localization of YAP from the nucleus to the cytoplasm (Wennmann et al., 2014). As known that G-protein-coupled receptor (GPCR) binding to the G protein subclass of receptors (Gα_*q/*__11_) activates YAP (Yu et al., 2012), and AT1R can activates YAP in HEK293 cells (Wennmann et al., 2014), we expected AT1R to did the same regulation in macrophages. However, AT1R stimulation with Ang II resulted in a decrease in the YAP/p-YAP ratio in macrophages. Recent studies have shown that Ang II can increase endothelial cell YAP phosphorylation (Fu et al., 2019) and that angiotensin-converting enzyme 2 activation induces pulmonary artery cell apoptosis by up-regulating YAP phosphorylation, thereby attenuating pulmonary vascular remodeling (Yan et al., 2019). Furthermore, study results have shown that after silencing YAP in macrophages, M1 related markers IL-12B, IL-1β, and TNF-α were up-regulated, and the expression of M1 markers CD68 and CD86 were enhance. In contrast, overexpression of YAP in macrophages increased the mRNA levels of M2 related markers IL-10, TGF-β and VEGF-A, and the expression of M2 markers CD163 and CD204 was significantly increased (Zhang et al., 2021). Therefore, our findings correlate well with these recent studies, as they indicate that Ang II can increase YAP phosphorylation and regulate macrophage activity.

Ang II induced YAP phosphorylation in macrophages by binding to AT1R, which leads to the polarization of more M1 macrophages and adhesion of macrophages to endothelial cells, ultimately leading to enhanced inflammation and endothelial cell injury. Therefore, it is likely that Ang II causes damage to endothelial cells by regulating YAP in macrophages. This suggests that promoting YAP dephosphorylation in macrophages could help prevent artray intimal injury.

Macrophages can be induced to polarize to an M1 or M2 state *in vitro*, and M1 macrophage polarization can be induced by upregulating YAP phosphorylation. Our observation that the decrease in the YAP/p-YAP ratio induced by Ang II binding to AT1R induced THP-1 cells to differentiate into M1 cells is supported by previous research showing that hepatocytes ectopically expressing an artificially activated YAP protein induce macrophage M2 polarization (Guo et al., 2017; Huang et al., 2017). M1 macrophages are a type of pro-inflammatory cell, therefore, we concluded that Ang II binding to AT1R promotes inflammation by regulating YAP phosphorylation, which is consistent with the changes in inflammation-related indicators (IL-6, MMP-9, and ET-1) observed in this study. Our results are consistent with the notion that patients with AD often exhibit inflammation (del Porto et al., 2010).

In animal studies, this study found that ARB treatment did reduce the incidence of AD and was more effective than simply lowering blood pressure. This is also consistent with the results of previous research by Ju et al. (2013) etc. The incidence of AD increased by Ang II does not depend on the increase in blood pressure. In order to study the effect of AT1R on macrophages in AD model, this study did not choose to use Ang II for modeling, but instead used BAPN to make AD model. At the same time, in order to eliminate the effect of blood pressure reduction on AD, the nifedipine experimental group was set up, and the blood pressure of this group was basically consistent with the telmisartan group by regular tail pressure measurement. At the same time, we verified in the cell experiments that nifedipine has no effect on YAP in macrophages, and the data is shown in the supplement. The results in animal experiments are also consistent with the results *in vitro* cell experiments. ARB does reduce the infiltration of M1 macrophages in the vessel wall and increases the number of M2 macrophages in the vessel wall. This suggests that ARB attenuates the pro-inflammatory effects of macrophages in the vessel wall by blocking AT1R, and enhances repair function. In the Western Blot results, the ratio of YAP/p-YAP decreased in the aortic tear part, and this ratio was significantly higher in the telmisartan-treated group than in the AD group. These results are consistent with those in the cell experiment. This suggests that AT1R plays an important role in regulating macrophage polarization and affecting the development of AD, and is highly likely to produce effects through YAP. More in-depth *in vivo* mechanism research is needed in the future.

There were some limitations to this study. First, More in-depth research is needed in the future. In this study, Western Blot was used to detect mixed samples in the aortic samples, so the change of YAP in macrophages in the aortic tear parts was not accurately detected. Second, we were unable to assess the effects of YAP overexpression on macrophage polarization and adhesion, as we failed to successfully overexpress YAP. Due to the limitations of experimental conditions and financial constraints, we were not been able to complete this part of the study.

Taken together, the findings reported here provide a new foundation by studying the integrated roles that AT1R and YAP play in regulating macrophage polarization and adhesion as driving forces of vascular intimal injury. Hence, the role of YAP in macrophages will be an important focus for further study, as it could be an interesting target for pharmacological intervention in vascular intimal injury in the future.

## Conclusion

AT1R induces YAP phosphorylation through binding to Ang II, and further promotes macrophages M1 polarization and adhesion to endothelial cells. ARB treatment effectively alleviates the above-mentioned pro-inflammatory phenomenon, reduces the incidence of AD in mice and affect macrophage polarization in mice aorta.

## Data Availability Statement

The raw data supporting the conclusions of this article will be made available by the authors, without undue reservation.

## Ethics Statement

The animal study was reviewed and approved by the Ethics Committee of Chinese People’s Liberation Army General Hospital.

## Author Contributions

XW, LC, YH, GS, and SJ conducted the cell experiments. XW, AM, LC, and YH conducted the animal experiments. YG and JL did the data collection and analysis. XW and AM wrote this manuscript. HZ, WG, LC, YG, DR, JL, SJ, GS, and YH revised the manuscript critically. All authors participated in the design, interpretation of the studies and analysis of the data, review of the manuscript, and approved for the manuscript submission.

## Conflict of Interest

The authors declare that the research was conducted in the absence of any commercial or financial relationships that could be construed as a potential conflict of interest.
