# Supplementary material for: AT1R Regulates Macrophage Polarization Through YAP and Regulates Aortic Dissection Incidence
Source: Front Physiol. 2021 Jul 9;12:644903. doi: 10.3389/fphys.2021.644903 (PMC8299470; doi:10.3389/fphys.2021.644903)
Supplement: Supplementary file 1 [file Data_Sheet_1.PDF]

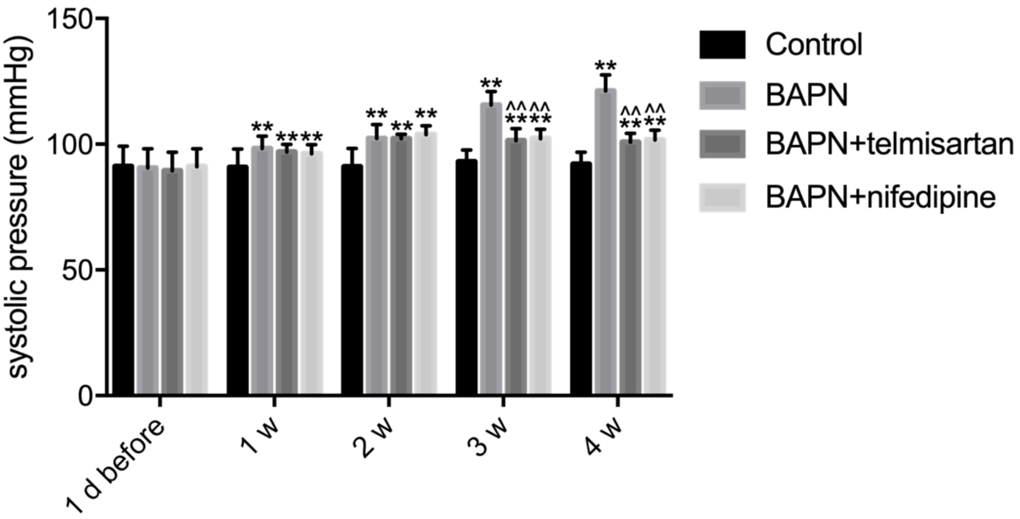

Figure : Tail-cuff tail pressure measurement of systolic blood pressure in mice. Histograms showing blood pressure changes of mice in each group 1 day before feeding, 1 week after feeding, 2 weeks after feeding, 3 weeks after feeding, and 4 weeks after feeding (n = 12; Mean and SD; One-Way ANOVA Analysis of variance; **, P <0.05 compared with the control group; ^^, P <0.05 compared with the BAPN fed group); Abbreviations: BAPN, β-aminopropionitrile.

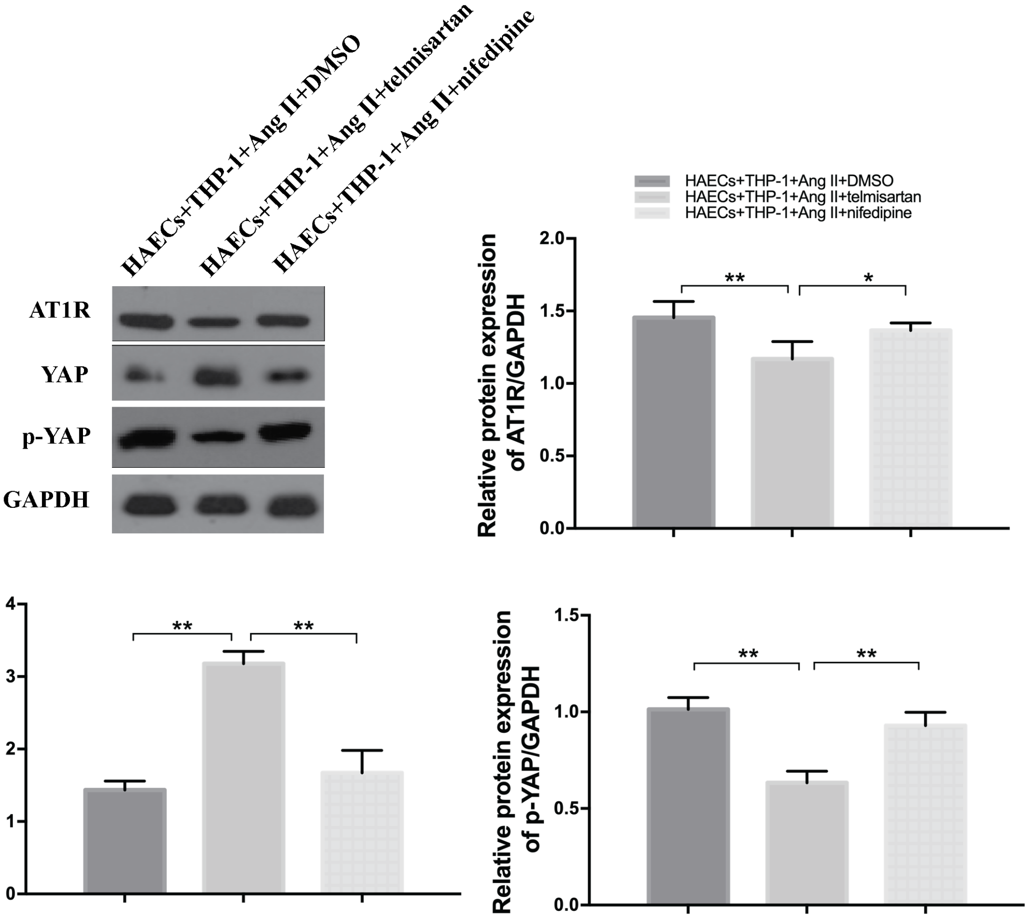

Figure: Western bolt analysis of six independent lysates from HAECS+THP-1 cells that were treated with Ang II and DMSO, treated with Ang II and telmisartan or treated with nifedipine. Ang II treatment up-regulated the content of AT1R and p-YAP, and this effect was alleviated

by AT1R siRNA transfection or telmisartan treatment. And, nifedipine has no influence on AT1R, YAP and p-YAP compared with DMSO treated group.
